# Delirium and Delirium Severity Predict the Trajectory of the Hierarchical Assessment of Balance and Mobility in Hospitalized Older People: Findings From the DECIDE Study

**DOI:** 10.1093/gerona/glab081

**Published:** 2021-03-16

**Authors:** Sarah Richardson, James Murray, Daniel Davis, Blossom C M Stephan, Louise Robinson, Carol Brayne, Linda Barnes, Stuart Parker, Avan A Sayer, Richard M Dodds, Louise Allan

**Affiliations:** 1 AGE Research Group, Translational and Clinical Research Institute, Faculty of Medical Sciences, Newcastle University, Newcastle upon Tyne, UK; 2 NIHR Newcastle Biomedical Research Centre, Newcastle University and Newcastle upon Tyne Hospitals NHS Foundation Trust, UK; 3 MRC Unit for Lifelong Health and Ageing at UCL, London, UK; 4 Institute of Mental Health, Division of Psychiatry and Applied Psychology, School of Medicine, Nottingham University, UK; 5 Population Health Sciences Institute, Faculty of Medical Sciences, Newcastle University, Newcastle upon Tyne, UK; 6 Cambridge Public Health, University of Cambridge, UK; 7 Centre for Research in Ageing and Cognitive Health, University of Exeter, UK

**Keywords:** Epidemiology, Hospital related, Physical function

## Abstract

**Background:**

Delirium is common, distressing, and associated with poor outcomes. Despite this, delirium remains poorly recognized, resulting in worse outcomes. There is an urgent need for methods to objectively assess for delirium. Physical function has been proposed as a potential surrogate marker, but few studies have monitored physical function in the context of delirium. We examined if trajectories of physical function are affected by the presence and severity of delirium in a representative sample of hospitalized participants older than 65 years.

**Method:**

During hospital admissions in 2016, we assessed participants from the Delirium and Cognitive Impact in Dementia study daily for delirium and physical function, using the Hierarchical Assessment of Balance and Mobility (HABAM). We used linear mixed models to assess the effect of delirium and delirium severity during admission on HABAM trajectory.

**Results:**

Of 178 participants, 58 experienced delirium during admission. Median HABAM scores in those with delirium were significantly higher (indicating worse mobility) than those without delirium. Modeling HABAM trajectories, HABAM scores at first assessment were worse in those with delirium than those without, by 0.76 (95% CI: 0.49–1.04) points. Participants with severe delirium experienced a much greater perturbance in their physical function, with an even lower value at first assessment and slower subsequent improvement.

**Conclusions:**

Physical function was worse in those with delirium compared to without. This supports the assertion that motor disturbances are a core feature of delirium and monitoring physical function, using a tool such as the HABAM, may have clinical utility as a surrogate marker for delirium and its resolution.

Delirium is an acute and fluctuating neurocognitive disorder, specifically affecting attention and level of arousal. Delirium is common and distressing, affecting 15% of older hospital inpatients (1), and is associated with poor outcomes, including mortality, institutionalization, and cognitive decline (2,3). Delirium remains poorly recognized and documented, resulting in worse outcomes, and there remains a lack of evidence regarding how best to monitor delirium longitudinally, including recognizing delirium resolution (1). Many of the cognitive tests currently used are not validated for repeated use, are burdensome, and rely heavily on testing cognition, which may be abnormal in people with dementia, with or without delirium (4). Therefore, there is an urgent need for reliable and reproducible methods of rapidly and objectively assessing for delirium.

Although delirium is primarily regarded as a cognitive disorder, motor disturbances have been proposed as a core feature of delirium and monitoring physical function has been suggested as a possible surrogate marker for delirium (5). However, previous work is limited by the use of measures of physical function which have significant floor effects in those with very limited or no mobility (6). The Hierarchical Assessment of Balance and Mobility (HABAM) consists of 3 domains: balance, transfers, and mobility, which are scored based on observation of the patient (7,8). The HABAM assesses across the spectrum of function, from fully dependent for pressure care to independent in transfers and mobility, and has been shown to be valid and reliable (9). Despite being shown to predict prevalent delirium when measured on admission, the HABAM has not previously been examined longitudinally in relation to delirium (10).

We aimed to describe the trajectories of HABAM over time in older, hospitalized patients and explore whether these trajectories varied by overall delirium status and delirium severity, along with exploring the effect of delirium on daily HABAM scores.

## Method

### Participants

This analysis uses data collected for the Delirium and Cognitive Impact in Dementia (DECIDE) study, which aimed to explore the association between delirium and cognitive function (3,11). The DECIDE study was nested within the Cognitive Function and Ageing Study II—Newcastle cohort (CFAS II-Newcastle), which provided a representative, population-based sample older than 65 years living within the catchment area of Newcastle upon Tyne Hospitals NHS Foundation Trust (12).

### Recruitment

From 5th January 2016 to 5th January 2017, we invited participants from the CFAS II-Newcastle to participate in DECIDE on admission to hospital. We were alerted to admissions by a Recurring Admission Patient Alert attached to the participants’ electronic records. For participants lacking capacity to consent, an appropriate personal consultee was requested to provide written confirmation of willingness to participate. Participants were excluded if they lacked capacity to consent and it was not possible to identify or contact an appropriate personal consultee, they were receiving end-of-life care, they were being isolated for infection control reasons, or they were expected to be in hospital for less than 24 hours. Once recruited, we recorded baseline data including age, sex, frailty using the Clinical Frailty Scale (13), comorbidity using the Cumulative Illness Rating Scale (14), and place of residence. Admissions were classified as the care of a medical or surgical team, with the latter divided into elective and emergency admissions. Early mobilization was routine on all wards in the study, including physiotherapy assessment when needed.

### Delirium and HABAM Assessments

Participants were seen daily for the DECIDE study, as far as possible, during their hospital admissions. Two research staff (S.R. and a specially trained research nurse) completed all of the assessments for the study. We ascertained delirium using a standardized approach based on the *Diagnostic and Statistical Manual of Mental Disorders*, fifth edition (DSM-5) criteria, described fully in the study protocol (11). In summary, the assessment combined objective testing of the participant, and information gained from informants (usually nurses, next of kin or clinical records), with structured observations made by the assessor. *Disturbance in attention* was evaluated using months of the year backwards and digit span, and arousal was recorded using the Observational Scale of Level of Arousal and the modified Richmond Agitation and Sedation Scale (15). *Disturbance in cognition* was evaluated using 3-item recall, 10 orientation questions, 3 stage commands, and any evidence of perceptual disturbances along with observations by the examiner during the interview. *Acute onset and fluctuating course*, change from baseline, and *evidence of underlying medical condition* were obtained from informant history from nursing staff, next of kin, and clinical records. Delirium presence or absence, along with delirium severity according to the Memorial Delirium Assessment Scale (MDAS) (16), was determined on each assessment.

We also recorded HABAM score on each assessment based upon observation by the assessor along with collateral history from nursing and physiotherapy staff. Participants were not specifically maneuvered for the purpose of the study but were observed throughout the interaction. Bedside clues such as the presence of zimmer frames and also signs at the patient bedside regarding levels of mobility (eg, WZF+1 = wheeled zimmer frame with assistance of one) were also noted. The medical notes were then reviewed for details regarding mobility during the preceding 24 hours, including any documentation by physiotherapists. If available, staff on the ward were asked how patients were mobilizing and transferring and whether any aids or assistance were required. Based on all of this information, we recorded scores for balance (out of 21), transfers (out of 18), and mobility (out of 26), with higher scores indicating better function (8).

### Statistical Analysis

In line with previous work, each HABAM component was transformed to lie in the range 0–1 by dividing the score obtained by the total score for each component (17). Also aligned to previous work, total HABAM score used in all analyses was calculated by summing the 3 individual items, giving a total score in the range 0–3, and then rescaled such that lower HABAM corresponded to better function. We used the previously developed cut points for the rescaled score: mild (≤1.25), moderate (1.26–1.74), and severe (≥1.75) functional impairment (17).

In instances where all 3 components were missing (*n* = 14 observations), no HABAM score was calculated. Where at least one other component was available (*n* = 4 observations), the missing component was imputed based upon the mean of available components, justified by the strong correlation seen previously between the 3 components (8).

We limited analysis to the first admission for participants during the study period with at least 2 documented HABAM scores. We also included only the first 14 days of admission due to the scarcity of data beyond this, as the majority of patients were discharged by this point. Characteristics of interest were first assessed for normality through use of the Shapiro–Wilks test. We assessed differences in HABAM scores, and other baseline variables, between those with and without delirium using student *t* test (parametric) and the Wilcoxon rank-sum test (nonparametric). The chi-squared test was used for assessing categorical variables.

We examined HABAM trajectories by fitting linear mixed-effects models, taking into account clustering at the level of individual patients. We tested for differences in the intercept and slope of HABAM trajectories between patients with and without delirium during their admission. We additionally divided those with delirium into thirds based on their maximum recorded MDAS score: categorized as mild, moderate, and severe. We then carried out a further linear mixed-effects model, with delirium subdivided into nonsevere (mild or moderate) and severe groups. Finally, in a separate model restricted to those with delirium during admission, we incorporated delirium diagnosis as a time-varying, binary predictor. This allowed for appraisal of the effect of delirium on daily HABAM score.

Patients who died during their selected admission were excluded in a sensitivity analysis. All statistical analyses were performed using R version 3.6.0 (www.r-project.org).

## Results

### Characteristics of Participants With and Without Delirium

The DECIDE study recruited 205 participants. Of these, 178 participants (53.9% female) had an admission with at least 2 valid HABAM scores and form the sample for the present analyses. The majority of participants (69.1%) were admitted under the care of a medical team. We found no differences in the age, sex, or frailty status of the 27 participants who did not have at least 2 valid HABAM scores.

Fifty-eight participants (32.6%) experienced delirium during the selected admission. Of those with delirium, 42 (72.4%) were diagnosed as such at first assessment and 47 (81.7%) had first diagnosis of delirium within 48 hours of their first assessment. No participants had delirium diagnosed beyond assessment Day 8 (please see Supplementary Figure 1). The characteristics of the sample are shown in Table 1. Those with delirium tended to have higher median HABAM scores (ie, worse performance) compared to those without and were frailer, older, and spent longer in hospital.

**Table 1.** Characteristics of Sample by Delirium Status

Variable	Total (*n* = 178)	Delirium (*n* = 58)	No Delirium (*n* = 120)	*p* Value^a^
Age, mean (*SD*)	82.3 (6.42)	84.8 (6.31)	81.1 (6.13)	.004
Sex: women, *n* (%)	96 (53.9)	32 (55.2)	64 (53.3)	.944
Living in 24-h care, *n* (%)	11 (6.2)	6 (10.3)	5 (4.2)	.109
Comorbidity score, mean (*SD*)	8.6 (4.4)	10.5 (4.2)	7.7 (4.2)	<.001
Clinical frailty score, median [IQR]	4 [3, 5]	5 [5, 6]	4 [3, 5]	<.001
Diagnosis of dementia, *n* (%)	19 (10.7)	16 (27.6)	3 (2.5)	<.001
Admission type, *n* (%):				.218
Medical	123 (69.1)	44 (75.9)	79 (65.8)	
Surgical (elective)	31 (17.4)	6 (10.3)	25 (20.8)	
Surgical (emergency)	24 (13.5)	8 (13.8)	16 (13.3)	
Length of admission, median [IQR]	7 [4, 14]	13 [9, 29]	5 [3, 8]	<.001
Number of assessments, median [IQR]	5 [3, 7]	7 [6, 11]	4 [2, 5]	<.001
Day of admission when HABAM first assessed, median [IQR]	1 [1, 2]	1 [1, 2]	2 [1, 2]	.131
HABAM total score^b^, median [IQR] and components:	0.99 [0.31, 1.93]	1.4 [0.76, 2.01]	0.76 [0.04, 1.69]	<.001
Balance component	0.33 [0.0, 0.67]	0.52 [0.33, 0.67]	0.33 [0.0, 0.52]	<.001
Mobility component	0.54 [0.04, 0.65]	0.54 [0.42, 0.65]	0.42 [0.04, 0.65]	<.001
Transfers component	0.0 [0.0, 0.61]	0.33 [0.0, 0.83]	0.0 [0.0, 0.61]	<.001

*Notes*: HABAM = Hierarchical Assessment of Balance and Mobility; IQR= interquartile range.

^a^ *p* Value from appropriate test for difference between delirium status. Where characteristic of interest reported as median [IQR], Wilcoxon rank-sum test was used; where mean (*SD*), student *t* test was used; and where categorical, chi-squared test was used. ^b^With higher values indicating worse function (see Method section).

### HABAM Trajectory in Those With and Without Delirium

**Figure 1.**
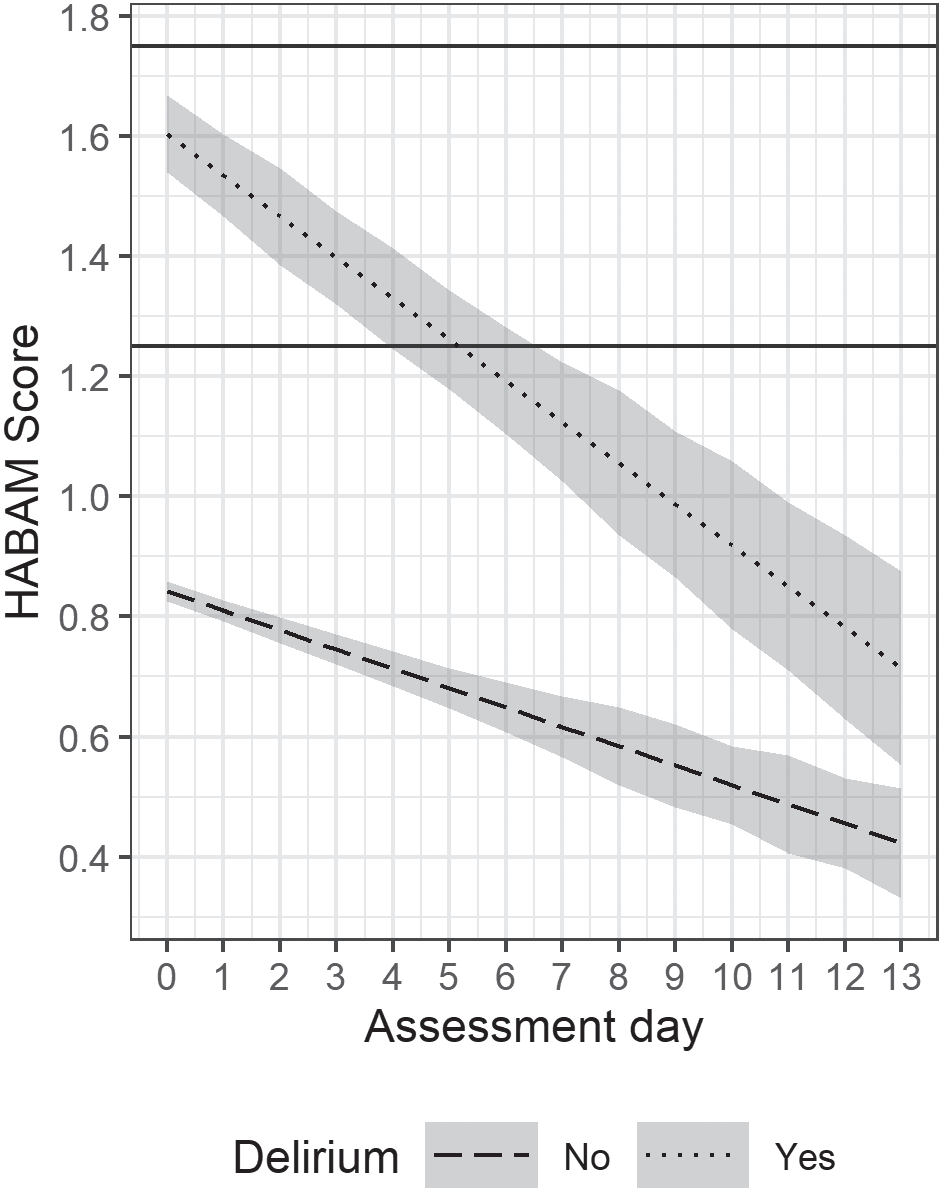
HABAM profiles during the first 14 d of admission in those with and without delirium. Predictions (with 95% confidence intervals) from linear mixed-effects model with fixed effects for assessment day, delirium diagnosis, and the interaction between the 2. Horizontal lines show the previously recommended cut points: ≤1.25 mild, 1.26–1.74 moderate, and ≥1.75 severe functional impairment.

Figure 1 illustrates the difference in baseline HABAM scores in those with and without delirium along with projections of improvement over the length of hospital stay, up to 14 days. The presence of delirium was associated with a higher HABAM score on first assessment, by an average of 0.76 points (95% CI: 0.49–1.04). HABAM scores improved on average over time. The mean daily improvement in those without delirium was 0.03 points (95% CI: 0.01–0.06). Delirium was associated with a nonsignificant additional daily improvement of 0.04 points (95% CI: −0.001, 0.074). Removing those who died during admission (*n* = 3) did not alter the findings.

### HABAM Trajectories Stratified by Delirium Severity

Those with severe delirium (*n* = 16, 9.0%) experienced more severe impairment in their physical function than those without delirium, with HABAM scores just over 1 point higher at first assessment (coefficient [95% CI]: 1.13 [0.72, 1.54]). This contrasted with those with nonsevere delirium, where the HABAM score was only slightly higher compared to those without delirium (coefficient [95% CI]: 0.62 [0.34, 0.90]). The improvement in HABAM score was more marked in the nonsevere delirium group, who showed an average daily improvement of 0.04 (95% CI: 0.02, 0.07) points greater than those without delirium, such that they had similar mean HABAM scores to the nondelirium group by day 14. These findings are also shown in Supplementary Figure 2.

### Change in HABAM Scores on Days With Delirium

**Figure 2.**
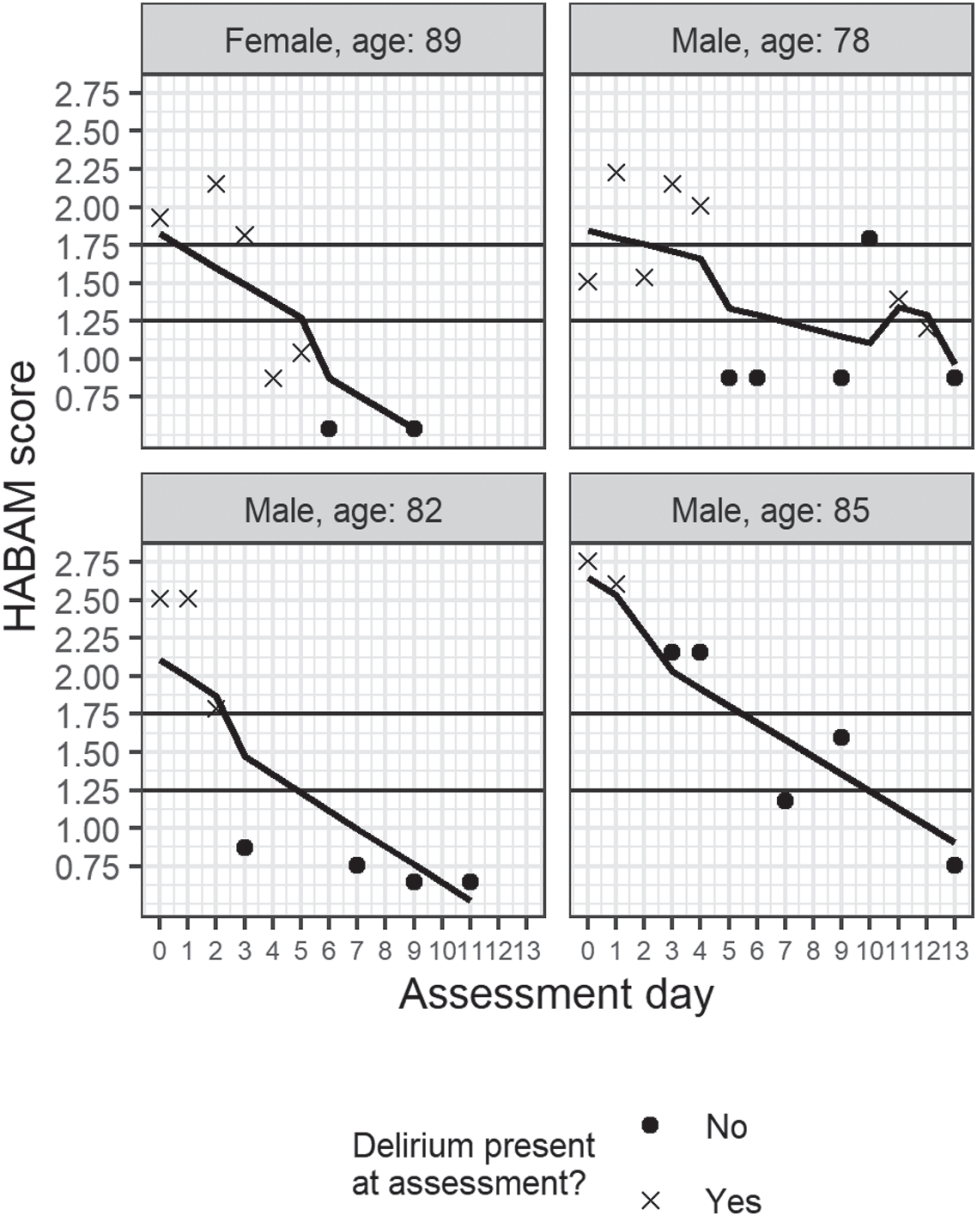
Examples of the day-by-day variation in total HABAM scores in individual participants. This figure shows examples of 4 patients who experienced delirium during their admission. The markers show the HABAM scores on the days it was assessed, with crosses and circles, indicating days when delirium was present and absent, respectively. The modeled lines are produced from a linear mixed model with delirium status (present or absent) as a time-varying covariate. Horizontal lines show the previously recommended cut points: ≤1.25 mild, 1.26–1.74 moderate, and ≥1.75 severe functional impairment.

When examining delirium status as a time-varying predictor, having delirium on a particular day was associated with a higher HABAM score (coefficient [95% CI]: 0.28 [0.15, 0.41]), indicating worse physical function, compared with days when delirium was not detected. To demonstrate this, Figure 2 shows HABAM profiles for 4 participants with fitted values from the longitudinal model. Improvement in HABAM score is seen as a participant moves from meeting criteria for delirium diagnosis to no delirium across assessments.

## Discussion

Our study shows that trajectories of physical function in older, hospitalized people, measured using the HABAM, differ in those with and without delirium. Those with delirium, and especially those with severe delirium, had worse physical function on admission. We also saw evidence of an improvement over time among those with delirium, although this appeared less marked in those with severe delirium.

A major strength of this work was that it was nested within CFAS II-Newcastle, a representative sample, including those with and without dementia. A further strength was the prospective delirium ascertainment using a standardized approach based on DSM-5 criteria, which has subsequently been replicated in other studies (18). Despite very low rates of missing data, a limitation of our study was that not all participants were seen every day due to participant refusal, illness, or study capacity, with only 2 researchers collecting data. However, a median number of assessments of 5 and a median length of stay of 7 days demonstrate that the majority of participants were seen near daily during admission. A further potential limitation was that assessors were not blinded to delirium status when evaluating physical function, as both were recorded at the same time. However, the HABAM is designed as a primarily objective measure and previous work has demonstrated high interrater and test–retest reliability (9). Finally, we did not have information on patients’ HABAM score prior to admission which would have allowed us to place any improvement in the context of a patient’s baseline mobility.

In line with previous work, we have shown that people with delirium have significantly worse physical function, measured using the HABAM, on first assessment (10). We have additionally shown that those with severe delirium have even greater derangement of their physical function. These findings suggest that measuring physical function, using the HABAM, may provide a useful surrogate marker for delirium, although further research is required. Additionally, our findings, which extend previous cross-sectional studies by using repeated measures of the HABAM, reveal that the trajectory of physical function in those with delirium tended to improve over 14 days, especially among those with nonsevere delirium which improved to a level similar to that seen in those who never had delirium. This improvement is likely to reflect delirium resolution, supported by our time-varying analysis, showing that physical function is worse on days when delirium is present compared to absent. This also supports previous work demonstrating that mobility impairments correlated with delirium status (6). Put together, these findings support the assertion that motor disturbances are a core feature of delirium and may have utility in differentiating those with and without delirium and in defining delirium resolution (5). We have also demonstrated that the HABAM, which has previously been shown to predict 30-day mortality, discharge destination, recovery time, and prevalent delirium (10,17), may be a useful tool for this purpose. Having been specifically designed to be used by clinicians at the bedside, it overcomes the floor effects associated with other measures. Further work is required to validate the HABAM specifically as a surrogate marker of delirium detection and its use to monitor for delirium resolution.

We understand very little about the complex relationships between physical and cognitive function, and the pathophysiology underlying these. We know that both predict poor outcomes, including mortality and institutionalization (8), and that each is a risk factor for the other (19), but further work is required to explore the fluctuations in physical function that occur at the time of delirium and whether monitoring physical function may provide an alternative strategy to improve delirium detection (20).

Trajectories of physical function during hospital admission, recorded using the HABAM, differ in older people with and without delirium. Our findings support the need for further research into whether monitoring physical function, using a tool such as the HABAM, may have clinical utility as a surrogate marker for delirium and delirium recovery during hospital admission. They also emphasize the importance of screening for delirium in those with mobility below their baseline on admission or a deterioration in their physical function during admission.

## Supplementary Material

glab081_suppl_Supplementary_Material
