# Supplementary material for: Delirium and Delirium Severity Predict the Trajectory of the Hierarchical Assessment of Balance and Mobility in Hospitalized Older People: Findings From the DECIDE Study
Source: J Gerontol A Biol Sci Med Sci. 2021 Mar 16;77(3):531–5. doi: 10.1093/gerona/glab081 (PMC8893191; doi:10.1093/gerona/glab081)
Supplement: glab081_suppl_Supplementary_Material [file glab081_suppl_supplementary_material.docx]

# Supplementary Material

## eFigure 1 – Assessment day of study in which participants with delirium first had a diagnosis of delirium made

Where day zero is the first day of assessment in the study.

## eFigure 2 – HABAM profiles during the first 14 days of admission in those with no delirium, non-severe delirium, and severe delirium


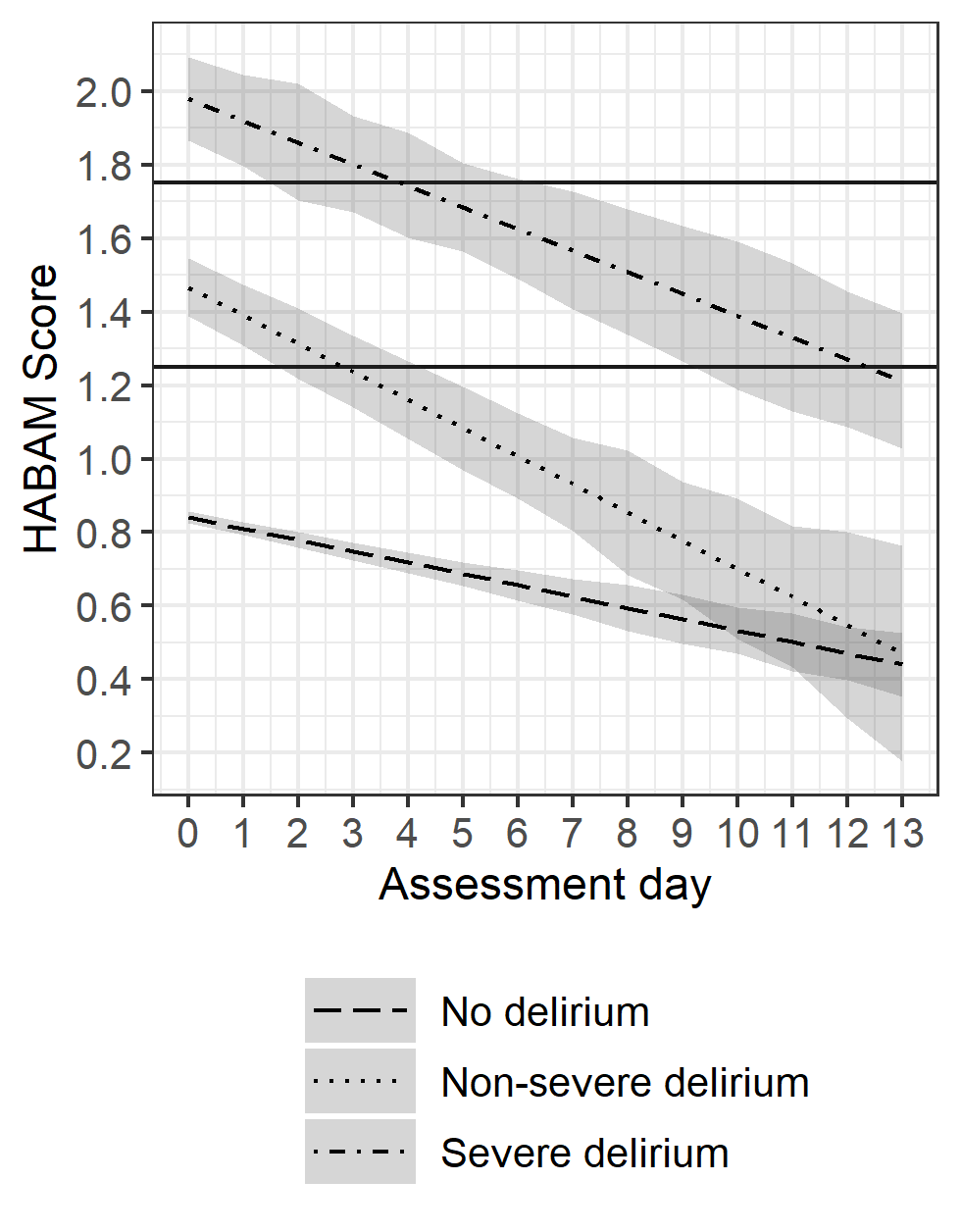


Predictions (with 95% confidence intervals) from linear mixed effects model with fixed effects for assessment day, delirium diagnosis (categorised as non-severe or severe based on peak MDAS score) and the interaction between the two. Horizontal lines show the previously recommended cut-points: <= 1.25 mild, 1.26 to 1.74 moderate and >= 1.75 severe functional impairment.
